# OctoChemDB: An Aggregated Database for Small Molecule Identification Using High-Resolution MS Data

**DOI:** 10.1021/acs.analchem.5c06761

**Published:** 2026-02-16

**Authors:** Ricardo Silvestre, Rémi Martinent, Laure Menin, Natalia Gasilova, Vincent Mutel, Cyril Portmann, Luc Patiny

**Affiliations:** † School of Engineering and Architecture, Institute of Chemical Technology, 27218 HES-SO University of Applied Sciences and Arts Western Switzerland, Fribourg 1700, Switzerland; ‡ Mass Spectrometry and Elemental Analysis Platform, Institute of Chemical Sciences and Engineering, École Polytechnique Fédérale de Lausanne, Lausanne 1015, Switzerland; § Inflamalps SA, Monthey 1870, Switzerland

## Abstract

High-resolution mass spectrometry (HRMS) is a cornerstone technology to dereplicate small molecules by comparing their MS spectral data to references in extensive chemical databases. However, most existing chemical databases lack robust support for processing spectral data or enabling direct *m*/*z*-based searches, limiting their usefulness for rapid compound identification. To address this, we developed OctoChemDB, a centralized database that aggregates and harmonizes chemical, biological, and spectral data from multiple open-access resources such as PubChem, MassBank, and GNPS. To make this data programmatically accessible, we implemented a REpresentational State Transfer Application Program Interface (REST API) that allows external tools and software to query the database using customizable parameters. This API serves as the core access point for developers and researchers to integrate OctoChemDB data into their own workflows and applications. As a practical demonstration of how the API can be used, we built a web application, available at https://octochemdb.cheminfo.org/, that enables users to perform *m*/*z*-based searches, predict molecular formulas, assess isotopic similarity, analyze fragmentation patterns, and retrieve associated literature and patents. This web interface serves as a user-friendly example of how the underlying database and API can be leveraged to accelerate small molecule identification. We illustrate the utility of the platform through case studies, including the identification of 3,4-methylenedioxymethamphetamine (MDMA) and caffeine, demonstrating its effectiveness in proposing structural hypotheses, matching experimental spectra with database entries, and streamlining dereplication workflows. The entire project, including source code, is available at https://github.com/cheminfo/octochemdb.

## Graphical abstract

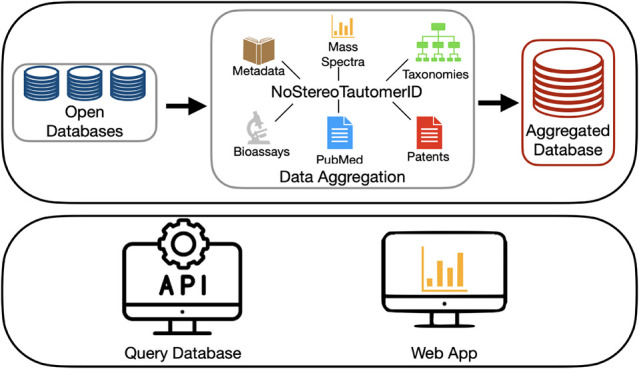

In the field of chemical research, identifying known molecules is essential for advancing studies in areas such as pharmaceuticals, environmental science, and materials development. Efficiently matching unknown spectra to known compounds within large chemical databases prevents redundant investigations and provides a foundation for further research. High-resolution mass spectrometry (HRMS) plays a key role in this process, offering the accuracy and resolving power needed to determine the mass-to-charge ratio (*m/z)* of ions in a sample allowing search in databases for the identification of compounds.^1^ While HRMS is widely used for identifying known compounds, it also plays a crucial role in dereplication, which involves distinguishing previously cataloged molecules from potential new ones. This application is particularly relevant in natural product research, where researchers must navigate complex mixtures to prioritize potential novel discoveries. Combining HRMS with advanced informatics tools can significantly improve the speed and efficiency of both identification and dereplication, enabling targeted searches based on *m*/*z* ratios.

However, conventional chemical databases often lack the necessary functionality to handle mass spectral data or support monoisotopic mass-based searches. Widely used databases such as PubChem^2^ and Lotus,^3^ for example, do not provide direct monoisotopic mass with mass accuracy search capabilities, complicating efforts to quickly match spectra to known compounds. Commercial software also presents challenges, including proprietary formats and delayed updates, limiting its utility in fast-paced research environments. In essence, the convergence of high-resolution mass spectrometry with advanced informatics tools introduces a new era in compound identification, aiming to make the process faster and more efficient.

Several specialized open-source tools have emerged to enhance the dereplication process using mass spectrometry data. MetFrag excels at annotating high-resolution MS/MS spectra by generating candidate structures from chemical databases and ranking them based on fragmentation patterns, making it highly effective for identifying unknown metabolites.^4^ The Global Natural Products Social Molecular Networking (GNPS) platform facilitates molecular networking, allowing researchers to leverage community-curated spectral libraries to identify natural products through MS/MS data comparison.^5^ MassBank serves as a robust open-access database of high-resolution mass spectra, enabling users to match experimental data against reference spectra for accurate compound identification.^6^ The Human Metabolome Database (HMDB) provides comprehensive metabolomic data, linking MS spectra with biological information to aid in understanding metabolic pathways.^7^

In this context, we developed OctoChemDB, an open-source, web-based platform that combines publicly available databases to provide an efficient way to search by monoisotopic mass with support for ionization mode and mass accuracy as well as by molecular formula and fragment ions, while simultaneously accessing associated literature, bioactivity data, and taxonomic information. OctoChemDB is designed to streamline compound identification processes by aggregating chemical, biological, and spectral data from multiple open-access resources. The platform includes a REST API that allows programmatic access to the database, enabling integration with external tools and workflows for small molecule identification.

OctoChemDB is open-source and available at https://github.com/cheminfo/octochemdb and https://octochemdb.cheminfo.org. It offers a browser-based environment that requires no installation. Calculations are performed using ChemCalc,^15^ and the development leverages the same technologies used in MSPolyCalc^16^ and CpHunter^17^ to enable mass spectrometry data processing and interactive data exploration. All computations and data handling are performed locally within the user’s browser, eliminating the need to upload spectra to remote servers. To improve usability, OctoChemDB includes several preloaded demo spectra that highlight its main functionalities. These examples allow users to explore the platform, become familiar with its analytical workflows, and assess its potential for use in their own research.

## Experimental Section

### Software and Libraries

OctoChemDB is hosted on a dedicated server running Ubuntu, utilizing Docker for containerized deployment to ensure modularity and reproducibility. The server is equipped with 24 CPU cores, 256 GB RAM, and 10 TB storage, providing sufficient computational power and storage capacity for the platform’s operations. The database is managed using MongoDB, a NoSQL system optimized for efficient data storage and retrieval. The backend relies on NodeJS using Fastify to serve as the API framework and web server.

### Case Study Materials

MDMA and caffeine standards were selected as case study of natural bioactive compounds. Samples were purchased from Sigma-Aldrich (references M-013 and PHR1009, respectively) and stock solutions were prepared at 1 mg/mL in methanol for MDMA and in H_2_O for caffeine. The solutions were further diluted with the spraying solution (CH_3_CN–H_2_O–HCOOH (50:49.9:0.1)) prior to infusion into the mass spectrometer. Acetonitrile UPLC/MS was purchased from Biosolve. Mass spectrometry analyses were performed on an Exploris240 FTMS instrument (Thermo Scientific, Bremen, Germany) operated in the positive mode coupled with a chip-based nano-ESI source (TriVersa Nanomate, Advion Biosciences, Ithaca, NY, U.S.A.) controlled by the Chipsoft 8.3.1 software (Advion BioScience). Samples were sprayed using an ionization voltage of +1.4 kV and a gas pressure of 0.30 psi. The temperature of the ion transfer capillary was 200 °C. FT-MS spectra were recorded in the 100–1500 *m*/*z* range with a resolution set to 120,000. The mass spectra were externally calibrated with the Pierce FlexMix calibration solution.

### Results and Discussion

In this section, we present the development process and key features of OctoChemDB. We first describe the creation and synchronization of the integrated chemical database, followed by the strategies used for data aggregation and taxonomy harmonization. We then detail the structure and functionalities of the REST API and illustrate how it enables programmatic access to the database. Finally, we demonstrate the practical application of OctoChemDB through the web interface, highlighting its main tools and capabilities using a selected case study.

### Selection of Open Databases

The selection of databases was based on identifying the largest database relevant to mass spectrometry data processing, literature, bioactivity information, and chemical structures. The selected databases are reported in Table 1.

**Table 1:** Open Databases Integration Summary^a^

Database	Entries integrated in OctoChemDB September 2025	Most recent publication or source URL
PubChem	118,238,289	^2^
PubMed	36,964,454	^8^
Lotus	276,517	^3^
Coconut	407,269	^9^
CMAUP	60,222	^10^
GNPS	412,190	^11^
NPASS	96,234	^12^
NP Atlas	33,372	^13^
MassBank	116,672	^6^
NCBI Taxonomies	2,571,078	^14^

a This table provides an overview of selected open databases, detailing the number of entries integrated from each database and the latest publication source.

These open-access resources provide extensive coverage of freely available information. As for PubChem, not only structural data but also 6.5 million biological test results and 31 million patents associated with the structures were obtained from it. Abstracts of articles related to molecules on PubChem were sourced from PubMed, while all available taxonomies associated with molecules, were obtained from NCBI Taxonomies. In the realm of natural products databases like Lotus, Coconut, CMAUP, NPASS, and NP Atlas served not only as sources for determining whether a molecule is a natural product but also provided information on biological activities and the taxonomy of the originating organisms. Finally, MassBank and GNPS contributed with an excellent data set of MS^2^ spectra, including information on experimental conditions.

### Synchronization and Aggregation of Open Databases

OctoChemDB was developed to maximize versatility while minimizing maintenance requirements. To this end, synchronization with each external database is handled by autonomous and robust plugins, enabling long-term automated updates without human intervention. This modular architecture allows for seamless expansion, new data sets can be incorporated by simply adding corresponding plugins, without modifying the core system. Such standardization enables centralized data management and facilitates the rapid development of new scientific tools without rebuilding foundational components.

The workflow was divided into two main phases: synchronization and aggregation, as illustrated in Figure 1. The synchronization phase encompasses both the initial database creation and its regular updating. Every 24 h, a cron job scheduler automatically launches the synchronization plugins. Each plugin independently checks whether the corresponding database requires updating. When an update is available, or if it is the first synchronization, the plugin downloads the relevant data. Once all necessary databases are synchronized, the aggregation process is automatically triggered to update the aggregated database. The system then returns to standby until the next scheduled cycle.

**Figure 1:**
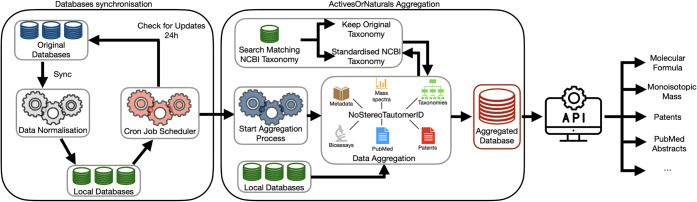
Data Synchronization and Aggregation Processes. During synchronization, plugins check for updates every 24 h. Updated data is downloaded and normalized using “NoStereoTautomerID” encoding. In aggregation, data elements are interconnected and consolidated into a unified document, with taxonomies normalized using NCBI standards. The aggregated database also provides a REST API that can be queried to retrieve data such as molecular formulas, monoisotopic masses, patents, PubMed abstracts, and other relevant information.

During synchronization, the data undergo processing, and structures are normalized to achieve the highest possible level of consistency between entries from different databases. A crucial step in this normalization is the generation of the so-called “NoStereoTautomerID”—a unique structure identifier grouping all stereoisomers and/or tautomers into a single entry,^18^ along with the corresponding data. This normalization is essential, as the same compound may appear in different tautomeric or stereochemical forms depending on the source, including undefined, racemic, or inconsistent absolute configurations. The NoStereoTautomerID is generated during the synchronization phase and is critical for the subsequent aggregation phase, as it allows the system to merge the entries from different databases into a single unified document.

During the aggregation process, additional labels are assigned when relevant. For instance, a “natural product” label is applied to structures originating from a recognized open database of natural products.

### Taxonomies Normalization

NCBI Taxonomies were adopted as the main standard, and to harmonize them, all taxonomies were aligned with the following levels: SuperKingdom, Kingdom, Phylum, Class, Order, Family, Genus, and Species. During the aggregation process, each taxonomy available for a selected compound was matched against the closest level of the NCBI taxonomies. When a match was found, the original taxonomy was supplemented with the standardized one. For example, if the taxonomy information for a given entry is known only from the family level to the species level and a match is found, the taxonomy tree would be reconstructed using the NCBI taxonomy. On the other hand, if no match occurred, the original taxonomy information is still preserved and normalized into the eight defined levels. In such cases, the levels that were not provided remain blank, while the known levels, such as species or superkingdom, are retained. This approach ensures that the structure of the taxonomy information remains consistent across all entries, regardless of whether a match with the NCBI taxonomy was found, while preserving any available user-provided information.

### Querying through API and Web Application Interface Overview

A REST API was developed for OctoChemDB to facilitate integration into external applications and provide programmatic access to the underlying data. The API follows OpenAPI specifications (formerly known as Swagger Specification)^19^ and mirrors the structure of OctoChemDB’s internal plugins, with each plugin exposing its own set of documented routes and customizable query parameters. The API is language-agnostic and can be accessed using any programming language that supports HTTPS requests, such as Python, R, or JavaScript. Detailed usage examples, complete documentation, and further resources can be found at https://octochemdb.cheminfo.org/documentation/.

The *Homepage Tab* of OctoChemDB API (Figure 2) serves as the starting point for MS spectra processing. By selecting the molecular ion peak, a list of possible molecular formulas will be generated. The calculation is based on user-defined parameters, including ranges of possible atoms, and mass accuracy. To further refine molecular formula selection, the *Similarity tab* evaluates isotopic pattern similarity between the experimental spectrum and simulated spectra^20^ from the candidate formulas generated in the *Homepage tab*. These formulas are ranked based on their similarity score, helping users prioritize the most probable molecular formula. The question mark button (Figure 2, point 5) provides direct access to the user documentation describing how to use OctoChemDB. In addition, export controls (Figure 2, point 6) allow users to download results as JSON files or copy them as tab-delimited tables suitable for spreadsheet software.

**Figure 2:**
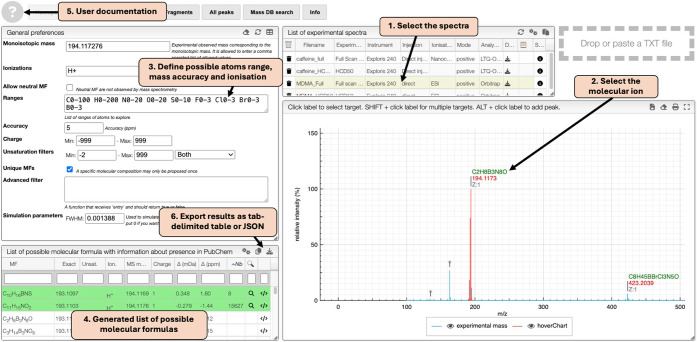
OctoChemDB Homepage Tab is a starting point for MS data processing. (1) Users first select the spectra. (2) They then select the monoisotopic mass corresponding to the molecular ion. (3) The calculation is guided by user-defined parameters, including atom ranges, ionization types, and mass accuracy. (4) Finally, a generated list of possible molecular formulas is provided. The question mark indicates the Help menu for the user. (5) The question mark button provides direct access to the user documentation describing how to use OctoChemDB. (6) Export controls allow users to download results as JSON files or copy them as tab-delimited tables suitable for spreadsheet software.

When MS/MS spectra are available, the *Fragments tab* helps prioritize molecular formula predictions by assessing the number of fragment ions assigned for each precursor ion’s molecular formula. For each fragment, possible molecular formulas are calculated based on accurate mass and evaluated to ensure elemental consistency with the precursor. The quality of the match is expressed as the percentage of fragment ions structurally compatible with the proposed precursor formula, enabling the selection of the most likely candidate.

The Mass DB Search tab (Figure 3) expands this analysis by allowing users to search selected fragments against literature-reported MS/MS spectra. This facilitates the identification of structurally related molecules even when exact matches are unavailable. By assuming that structurally similar compounds often fragment in similar ways, the tool supports the generation of structural modification hypotheses, such as the presence or absence of functional groups, e.g., methyl or hydroxyl. This feature also allows users to formulate hypotheses on potential substructures present in the unknown compound. When the majority of matched molecules share a specific substructure, it can be hypothesized that the same substructure may also be present in the unknown sample.

**Figure 3:**
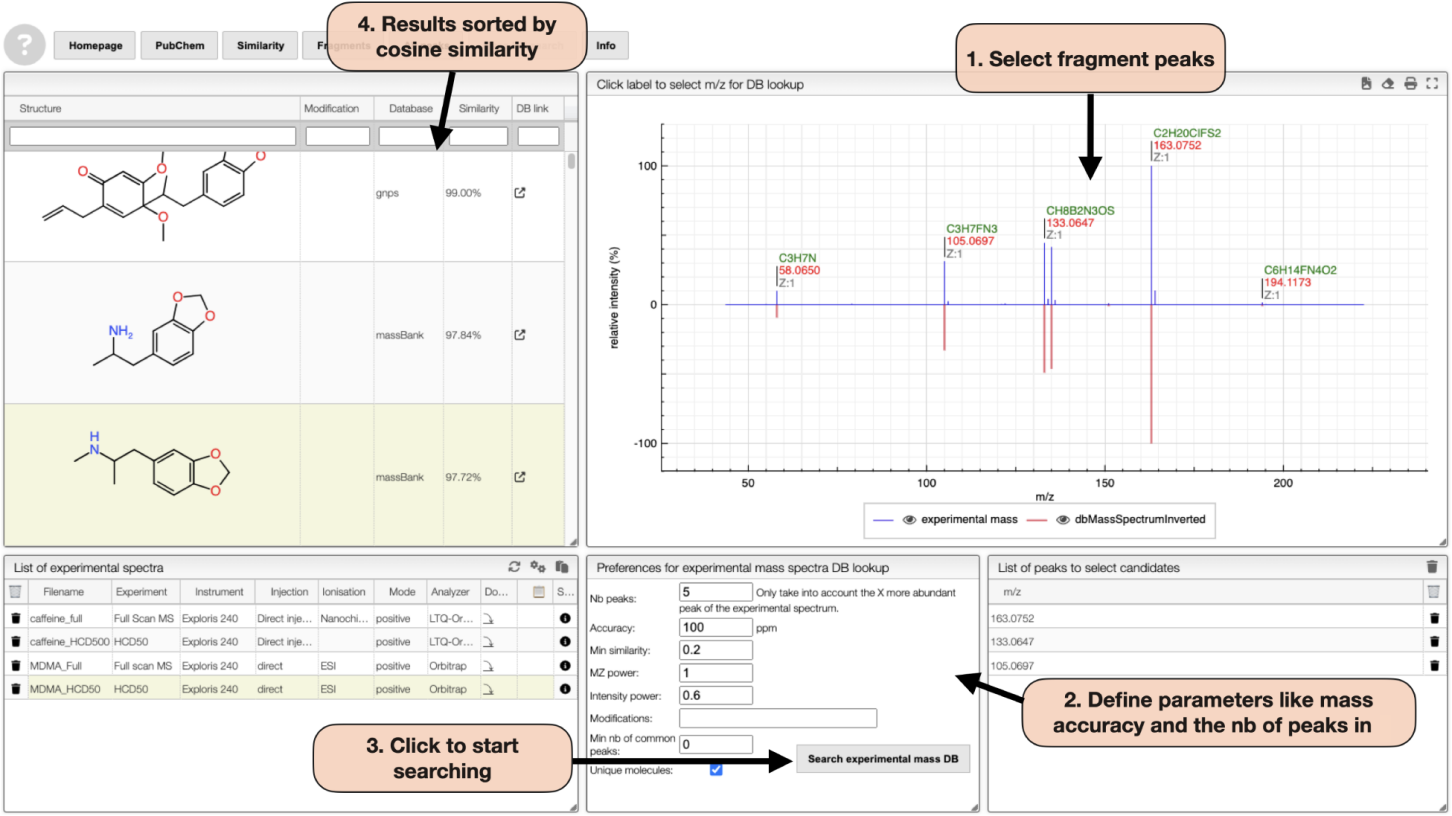
OctoChemDB Mass DB Search Tab allows expanding fragmentation analysis by searching selected fragment ions in literature-reported MS/MS spectra. (1) Users begin by selecting fragment peaks. (2) Parameters such as mass accuracy and the number of peaks are then defined. (3) A search can be initiated to query the database. (4) Results are sorted by cosine similarity, enabling the identification of structurally related compounds based on shared fragmentation patterns.

The *PubChem tab* facilitates the identification of molecular formula candidates without requiring manual specification of an atom range. The software automatically queries PubChem for molecular formulas that match the precursor ion’s *m*/*z*, taking into account the ionization type and user-defined mass accuracy. Only formulas associated with at least five structures in PubChem and corresponding to neutral molecules with no net charge are considered. This was implemented to avoid exotic or poorly characterized structures that can be found in PubChem. Molecular formulas with fewer than five known structures are more likely to represent rare, unstable, or artifactual compounds, which could introduce noise or bias into the analysis. By applying this threshold, we aim to ensure a more robust and representative data set of commonly observed small molecules. Retrieved formulas are displayed in a ranked list, with those linked to known bioactive compounds or natural products highlighted in green, allowing users to quickly recognize potentially relevant candidates. Furthermore, the PubChem results can be filtered based on functional groups or substructures, which is particularly advantageous when only the chemical class or a partial structural feature of the compound is known.

The *Mass Spectra Matching tab*, accessible via the flask icon within the PubChem tab, enables rapid comparison of experimental MS/MS spectra against literature-reported spectra. Matches are ranked based on cosine similarity, helping users to identify compounds with similar fragmentation patterns from existing spectral databases.^21^

The *Literature Review tab* (Figure 4), accessible by clicking the biohazard button in the PubChem tab, provides tools for exploring natural products and bioactive compounds associated with the selected molecular formula. Once a structure is chosen, a detailed panel opens displaying related PubMed abstracts, bioactivity data from PubChem bioassays, patents, and the taxonomic classification of the source organism. This integration allows researchers to efficiently access both spectral and biological information, supporting the dereplication and identification of small molecules.

**Figure 4:**
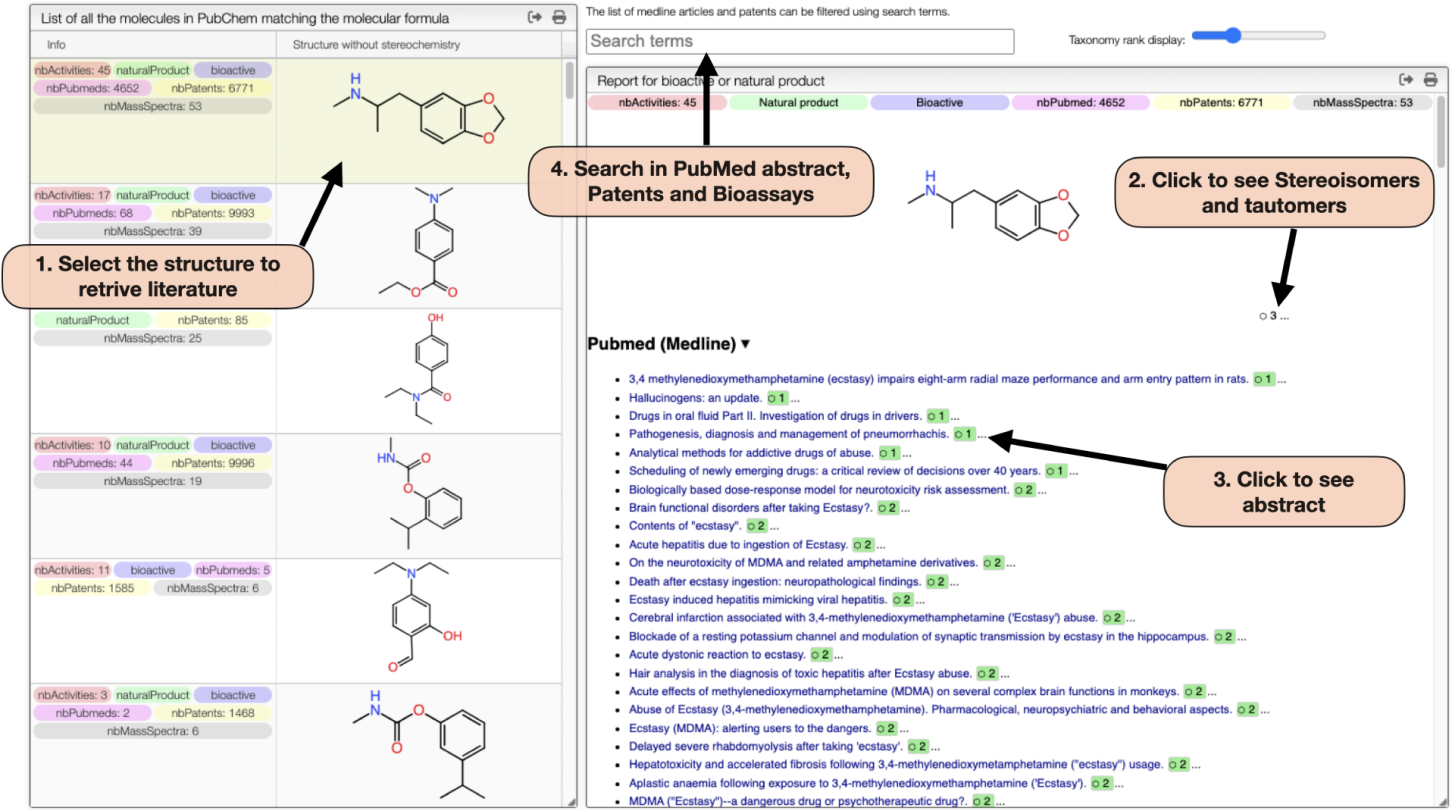
OctoChemDB Literature Review Tab allows exploring natural products and bioactive compounds associated with the selected molecular formula from PubChem. (1) Upon selecting a structure, the literature review panel retrieves linked information. (2) Users can also explore stereoisomers and tautomers of the selected compound. (3) PubMed abstracts can be directly accessed by clicking on the links. (4) Searches cover PubMed abstracts, bioactivity assay data, patent information, and the taxonomic classification of the source organism. This integration facilitates efficient access to chemical, biological, and bibliographic data for compound dereplication. Green numbers indicate the number of molecules discussed in each article, enabling users to prioritize articles that are more specifically focused on the molecule of interest.

### Case Study

In this article only the case study of MDMA will be presented while the caffeine case study can be found in the Supporting Information. The 3,4-methylenedioxymethamphetamine or MDMA, also known as ecstasy or Molly, is a synthetic psychoactive drug known for its euphoric effects. It alters mood and perception by increasing serotonin, dopamine, and norepinephrine levels in the brain.^22^ Due to its dangerous side effects and law enforcement interest in identifying it, it was chosen as a suitable case study of a synthetic bioactive compound to demonstrate the capabilities of OctoChemDB.

#### Molecular Formula Determination

The experimental mass^1^ spectrum of the sample showed a main ion at 194.1173 *m*/*z*. On the *Homepage Tab*, to generate the list of candidate molecular formulas, the ionization type was defined as [M + H]^+^, an accuracy of 5 ppm was set, and the range of elements was defined as follows: C_0–100_, H_0–200_, N_0–20_, O_0–20_, S_0–10_, F_0–3_, Cl_0–3_, Br_0–3_, B_0–3_. Once the ion at 194.1173 *m*/*z* was selected, 78 candidate molecular formulas were generated, two of them having reported structures on PubChem (green line background). To discriminate between the two possible candidate formulas, in the *Similarity Tab*, the similarity of the isotopic pattern was calculated, resulting in 92.51% for the molecular formula C_10_H_16_BNS and 99.23% for C_11_H_15_NO_2_. In the Fragments Tab, the MS/MS HCD spectrum of the sample displayed a percentage of fragments assigned of 16.64% for C_10_H_16_BNS and 91.90% for C_11_H_15_NO_2_. Finally, on the *PubChem Tab*, only the two candidate formulas are shown since they are the only ones that have reported structures on PubChem, and only C_11_H_15_NO_2_ had reported bioactive and/or natural structures, which could be useful in the case of unknown samples. With all the data combined, the molecular formula C_11_H_15_NO_2_ was selected as the most probable one.

#### Fragmentation Patterns Matching

On the Mass DB Search Tab, the ions 105.0697 *m*/*z*, 133.0647 *m*/*z*, and 163.0752 *m*/*z* were selected to search literature MS/MS spectra, resulting in 9 structures found. It was observed that 7 of them had 1,3-Benzodioxole as a substructure (see Figure 3), leading to the hypothesis that the sample might have the same substructure.

#### Literature Review

From the PubChem Tab, Literature Tab and the molecular formula C_11_H_15_NO_2_, the 106 structures can be displayed by clicking on the biohazard button. Among them 68 structures are bioactive. Finally, the substructure 1,3-Benzodioxole hypothesized before from MS/MS spectrum was used to further filter the list, narrowing it down to a single compatible structure: 3,4-Methylenedioxymethamphetamine, also known as MDMA. For this molecule, 45 bioassays with positive results are reported, along with 4591 PubMed abstracts and 6771 patents abstracts available for performing a text search.

#### Mass Spectra Database Matching

Under the Mass DB Search Tab, experimental MS/MS spectra can be systematically compared to literature-reported spectra. The database search is initiated using the precursor ion’s mass-to-charge ratio (*m*/*z*), followed by spectral alignment and similarity scoring on selected fragment ions based on cosine similarity metrics. As illustrated in Figure 4, the experimental MS/MS spectrum of MDMA acquired using HCD activation exhibits a high degree of overlap with the reference spectrum, yielding a cosine similarity score of 97.33%, which strongly supports the proposed identification. In contrast, all other candidate structures displayed similarity scores below 4%, indicating poor spectral concordance.

## Conclusions

OctoChemDB addresses a key challenge in small molecule identification by providing an efficient, web-based tool that integrates high-resolution mass spectrometry data with access to literature and open-access databases. By streamlining the dereplication process, it allows researchers to effectively distinguish between known compounds and potential new discoveries. The platform’s features—such as the generation of candidate molecular formulas, isotopic pattern matching, and MS/MS fragment analysis—are particularly valuable for handling complex data sets in a straightforward manner.

Through case studies involving compounds like MDMA and caffeine (see Supporting Information), OctoChemDB demonstrates its capability in rapidly identifying molecular formulas and proposing structural hypotheses. Additionally, the integration of bioactivity data, patent information, PubMed articles, and taxonomic classifications enhances the contextual understanding of each compound. The data used in these case studies is available on the OctoChemDB platform, allowing users to explore the tool and familiarize themselves with its functionalities. This comprehensive approach enables users to explore relevant scientific literature and biological activity data, all within a single platform.

## Supplementary Material



## Notes

Detailed case study demonstrating the dereplication of caffeine using the OctoChemDB web application; file includes a step-by-step methodology; input mass spectrometry data; screenshots of the user interface; dereplication results; discussion of database query results; material provides insight into the practical application of OctoChemDB for small molecule identification; File: Caffeine_Dereplication_Case_Study (PDF)
