# Supplementary material for: OctoChemDB: An Aggregated Database for Small Molecule Identification Using High-Resolution MS Data
Source: Anal Chem. 2026 Feb 16;98(8):6102–8. doi: 10.1021/acs.analchem.5c06761 (PMC12961639; doi:10.1021/acs.analchem.5c06761)
Supplement: Supplementary file 1 [file ac5c06761_si_001.pdf]

OctoChemDB: An Aggregated Database for Small Molecule Identification Using High-Resolution MS Data

Ricardo Silvestre[λ,π,‡], Rémi Martinent[λ], Laure Menin[π], Natalia Gasilova[π], Vincent Mutel[θ], Cyril Portmann[λ], Luc Patiny[π,*,‡]

[λ] School of Engineering and Architecture, Institute of Chemical Technology, HES-SO University of Applied Sciences and Arts Western Switzerland, Fribourg, 1700, Switzerland

[π]Mass Spectrometry and Elemental Analysis Platform, Institute of Chemical Sciences and Engineering, École Polytechnique Fédérale de Lausanne, Lausanne, 1015, Switzerland

[θ]Inflamalps SA, Monthey, 1870, Switzerland

*Email: luc.patiny@epfl.ch

[‡]R.S. and L.P. contributed equally to this work

Table of Contents

Case Study: Caffeine ***S2***

Introduction S2

Molecular Formula Determination S2

Similarity Evaluation S3

Fragmentation Matching S4

Fragmentation Patterns Matching S5

PubChem Tab S6

Literature Review S7

Mass Spectra Database Matching S8

Conclusion S9

Case Study: Caffeine

Introduction

Caffeine (1,3,7-trimethylxanthine) is a well-known stimulant widely consumed in coffee, tea, and various soft drinks. Its presence in natural and synthetic mixtures makes it a relevant candidate for dereplication. This case study illustrates how OctoChemDB can be used to rapidly identify caffeine from MS and MS/MS data by integrating molecular formula prediction, spectral similarity analysis, fragment ion assignment, and literature-based contextualization.

Molecular Formula Determination

To initiate dereplication, the experimental mass spectrum of the sample was uploaded to OctoChemDB. The main observed ion was located at 195.0873 m/z (see Figure S1). The ionization mode was set to [M+H]+, with a mass accuracy of 5 ppm. The allowed element ranges were defined as:

- $C_{0\text{-}100}$, $H_{0\text{-}200}$, $N_{0\text{-}20}$, $O_{0\text{-}20}$, $S_{0\text{-}10}$, $F_{0\text{-}3}$, $Cl_{0\text{-}3}$, $Br_{0\text{-}3}$.

Upon selection of the peak, 9 candidate molecular formulas were generated. Among them, two had corresponding structures found in PubChem. The formula $C_8H_{10}N_4O_2$ and $C_8H_{18}OS_2$ were selected as candidates since they are the only two with known structures reported on PubChem, to discriminate between the most probable, the similarity of isotopic pattern and the fragments matching will be done.

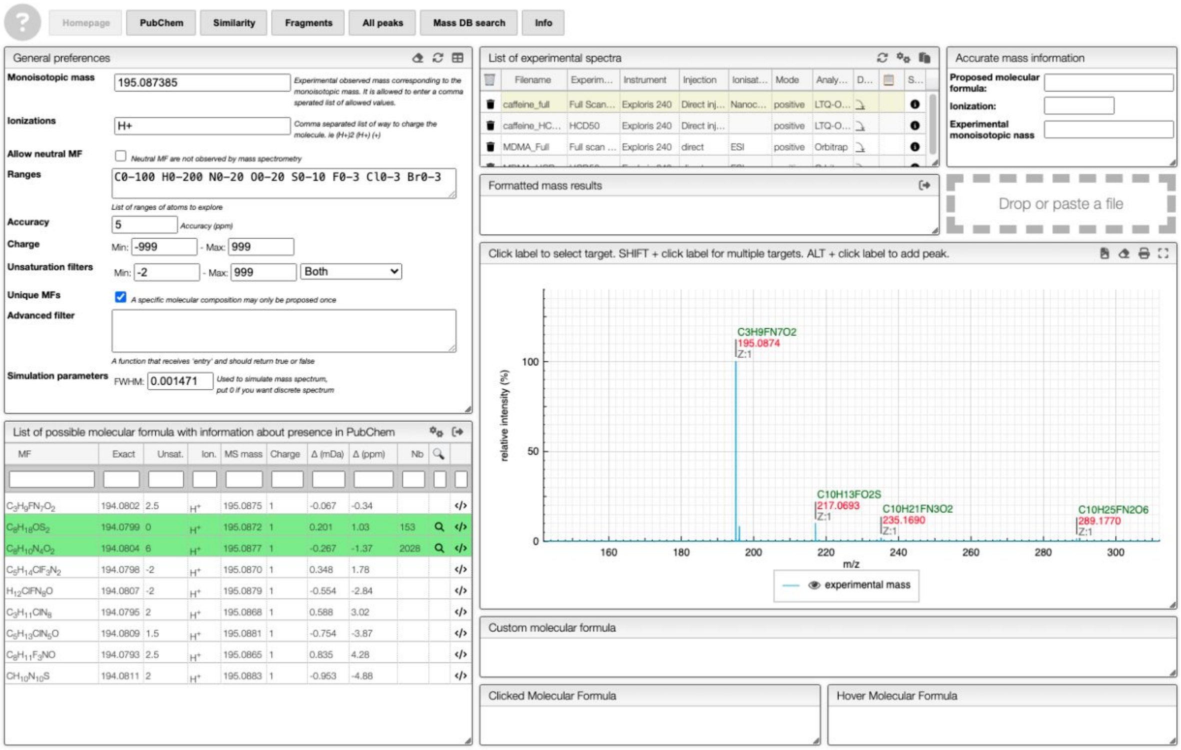

Figure S1: *Homepage Tab showing input parameters (ion type, ppm accuracy, elements range) and the list of generated candidate molecular formulas based on the selected m/z peak.*

Similarity Evaluation

In the Similarity Tab, isotopic pattern similarity scores were calculated for the top candidate molecular formulas. The formula $C_8H_{10}N_4O_2$ showed an isotopic pattern match of 99.57%, outperforming another plausible candidate, $C_8H_{18}OS_2$, which had a score of 92.34% (see Figure S2).

This high degree of isotopic match further supported the selection of $C_8H_{10}N_4O_2$ as the likely molecular formula of the unknown compound.

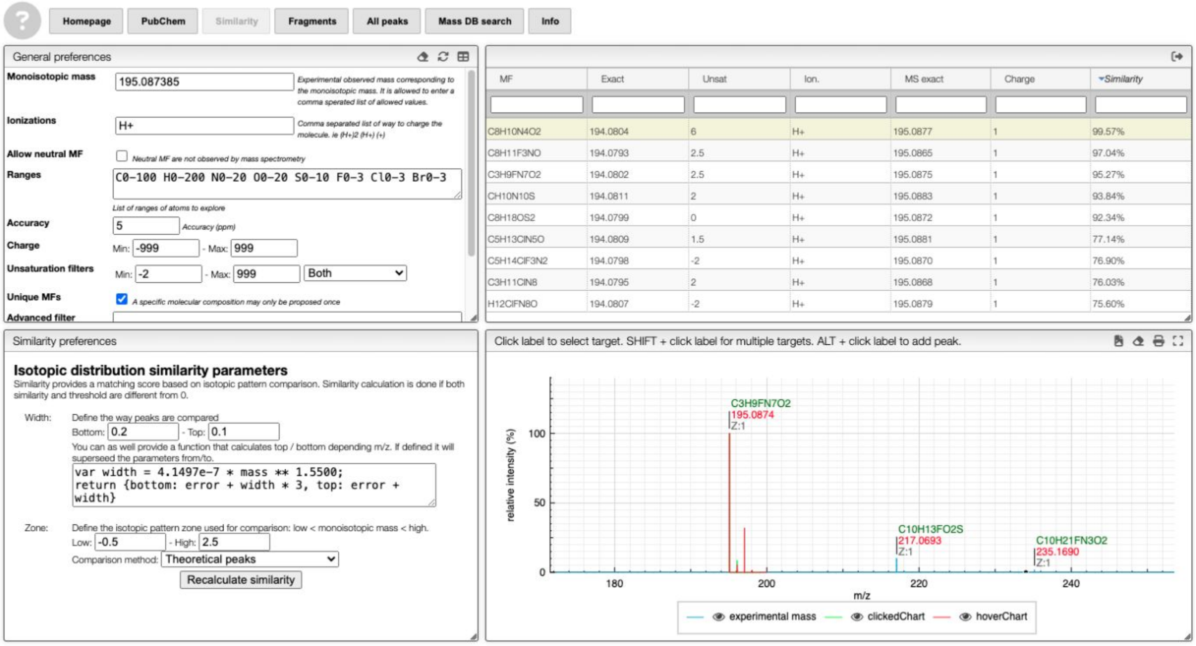

Figure S2: *Similarity Tab displaying isotopic pattern similarity scores for candidate molecular formulas.* $C_8H_{10}N_4O_2$ *shows the highest similarity (99.57%) compared to* $C_8H_{18}OS_2$ *(92.34%).*

Fragmentation Matching

When the MS/MS HCD spectrum of the sample was selected in the Fragments Tab, the software automatically annotated the fragment ions and calculated the percentage of fragments that matched theoretical predictions for each candidate molecular formula.

For $C_8H_{10}N_4O_2$, a match score of 92.76% was obtained, significantly higher than that of the alternative formula $C_8H_{18}OS_2$, which showed a match score of only 45.79%. These results, illustrated in Figure S3, provided strong support for $C_8H_{10}N_4O_2$ as the most likely molecular formula of the unknown compound.

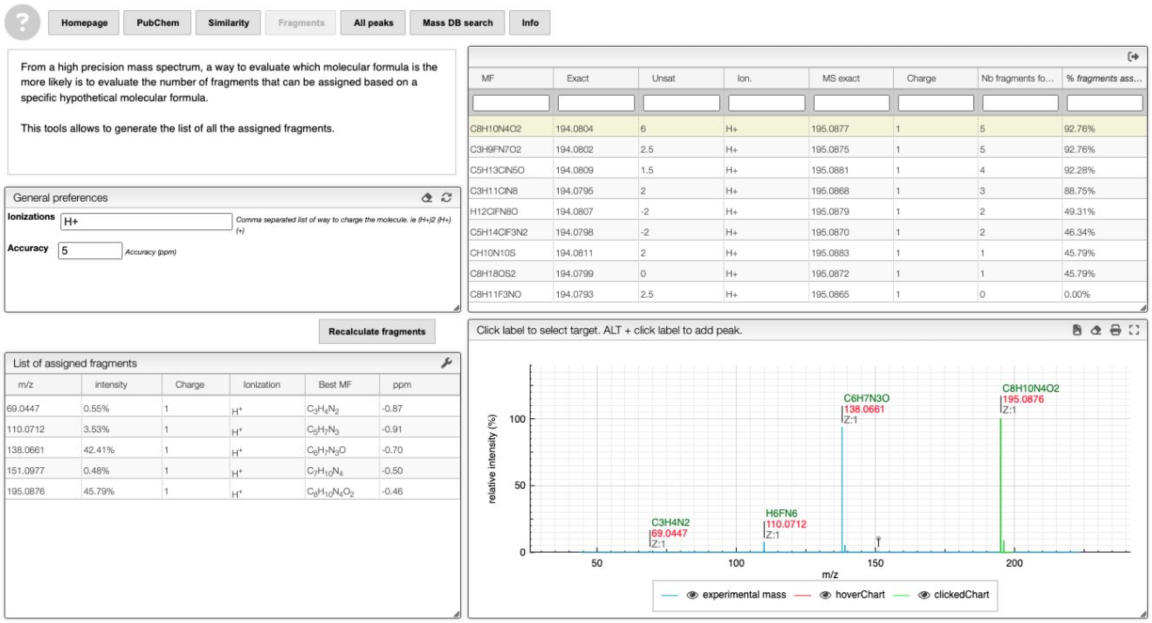

Figure S3: *Fragments Tab showing MS/MS HCD spectrum fragment matching results.* $C_8H_{10}N_4O_2$ *demonstrates a 92.76% fragment match score, while* $C_8H_{18}OS_2$ *scores only 45.79%.*

Fragmentation Patterns Matching

In the Mass DB Search Tab, the fragment peaks at 69.0447 m/z, 110.0711 m/z, and 138.0661 m/z were selected to search for matching MS/MS spectra in the literature (see Figure S4). This search returned 26 candidate structures, of which 19 contained an Imidazole substructure. Based on this prevalence, it was hypothesized that the unknown compound likely shares the same substructure.

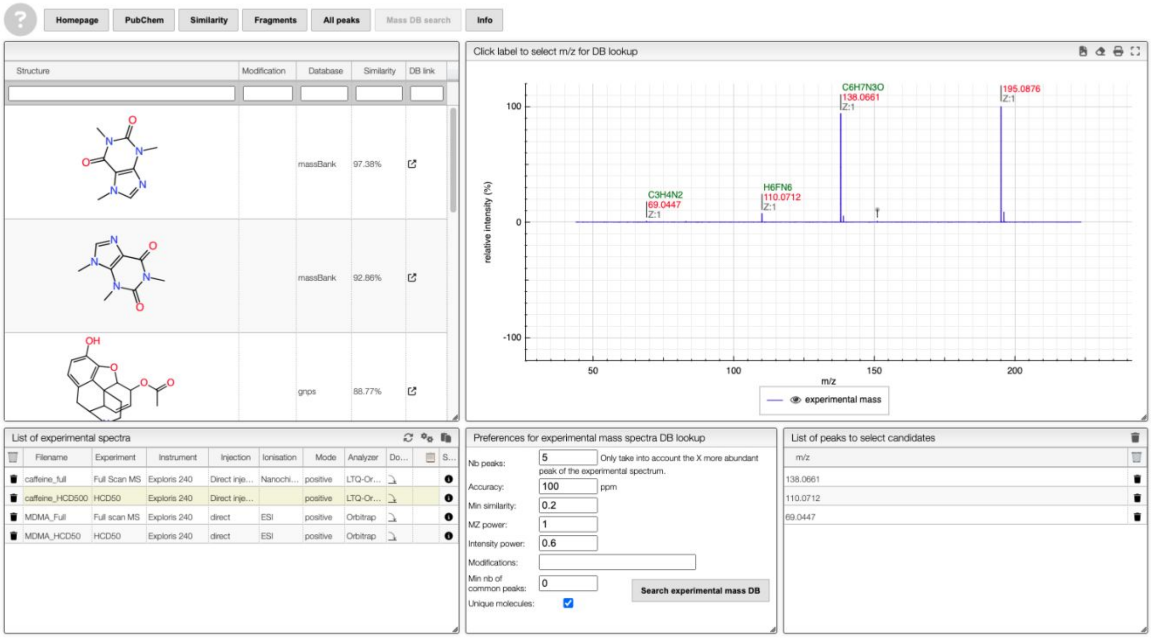

Figure S4: *Mass DB Search Tab showing the selection of diagnostic fragment ions (69.0447, 110.0711, and 138.0661 m/z) and the list of 26 matching structures, 19 of which contain an Imidazole substructure.*

PubChem Tab

In the PubChem Tab, the candidate molecular formula $C_8H_{10}N_4O_2$ was associated with 2,028 known structures in the PubChem database. Among these, 11 were reported as natural products and 23 as bioactive compounds, with possible overlap between the two categories (see Figure S5). This information provided an initial indication of the compound's relevance as a natural bioactive molecule.

Additional data can be accessed directly from the interface: the Literature Tab can be opened by clicking on the biohazard icon, while the available MS/MS spectra (535 entries) can be accessed by clicking on the flask icon. These tools allow users to explore biological activity and spectral data for each structure directly within the platform.

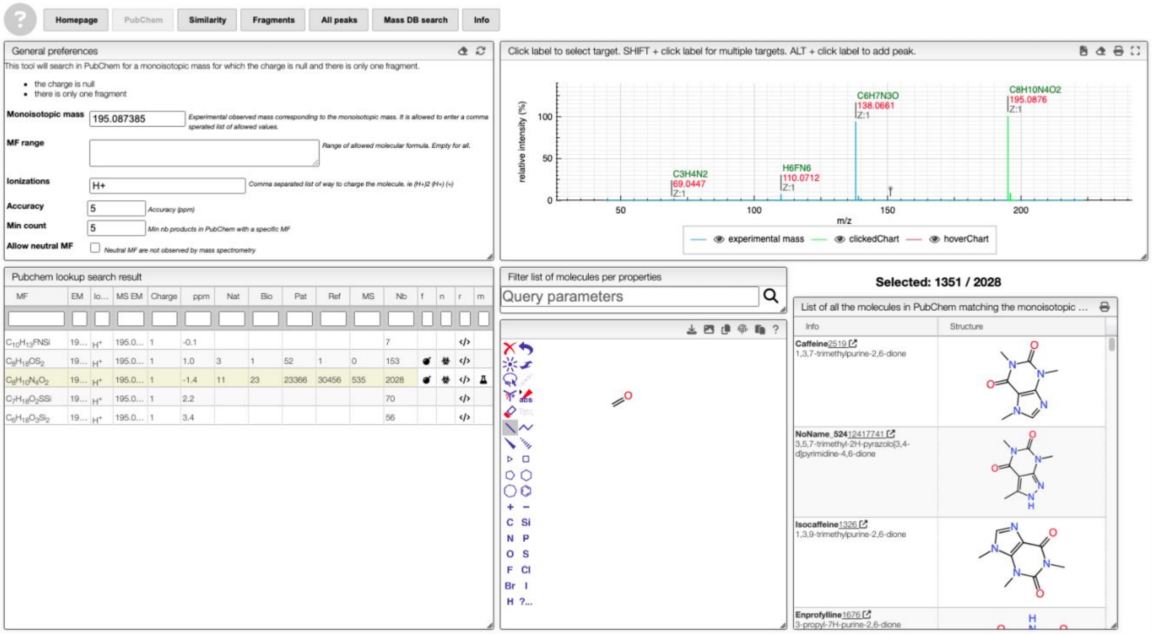

Figure S5: *PubChem Tab displaying molecular formula $C_8H_{10}N_4O_2$ with 2,028 associated structures, highlighting counts of natural and bioactive compounds, and access to MS/MS spectra and literature links.*

Literature Review

On the PubChem Tab, the Literature Tab for the molecular formula $C_8H_{10}N_4O_2$ was opened by clicking on the biohazard button, revealing 30 associated structures (see Figure S6). Since the sample was known to contain a bioactive compound, the list was narrowed down to 23 structures with reported biological activity.

However, this number was still too large to confidently identify the compound. To further refine the selection, the previously hypothesized Imidazole substructure, inferred from fragmentation pattern analysis, was used as a filtering criterion. This led to a shortlist of only three compatible structures: Caffeine, Enprofylline, and 8-Methyltheophylline.

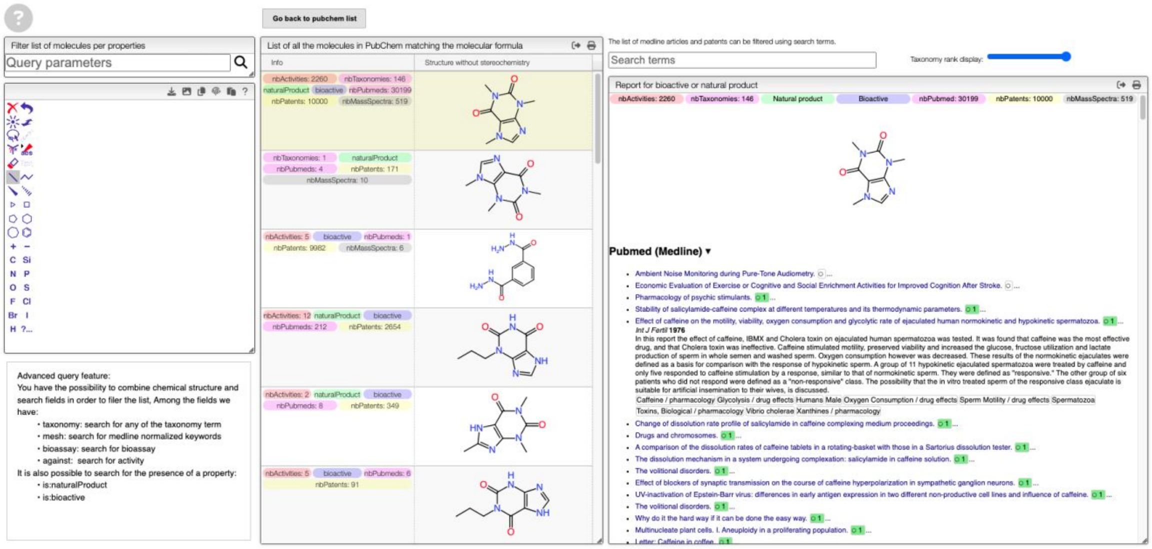

Figure S6: *Literature Tab interface showing bioactive structures associated with $C_8H_{10}N_4O_2$. The Imidazole substructure filter reduced the list to Caffeine, Enprofylline, and 8-Methyltheophylline.*

Mass Spectra Database Matching

To confirm the identity of the compound, the MS/MS HCD 50 experimental spectrum was matched against reference spectra available in the literature. This was done through the PubChem Tab by clicking on the flask icon, which opens the MS/MS spectral database for candidate structures.

The search used the precursor ion m/z to retrieve relevant spectra and calculated the cosine similarity between the experimental and reference spectra. The top three hits were (see Figure S7):

- Caffeine, with a similarity score of 99.98%
- Isocaffeine, with 96.27%
- 1,3-Benzenedicarboxylic acid, dihydrazide, with 82.24%

The extremely high similarity score for Caffeine, supported by isotopic pattern and fragment matching, confirmed it as the most probable identity of the unknown compound.

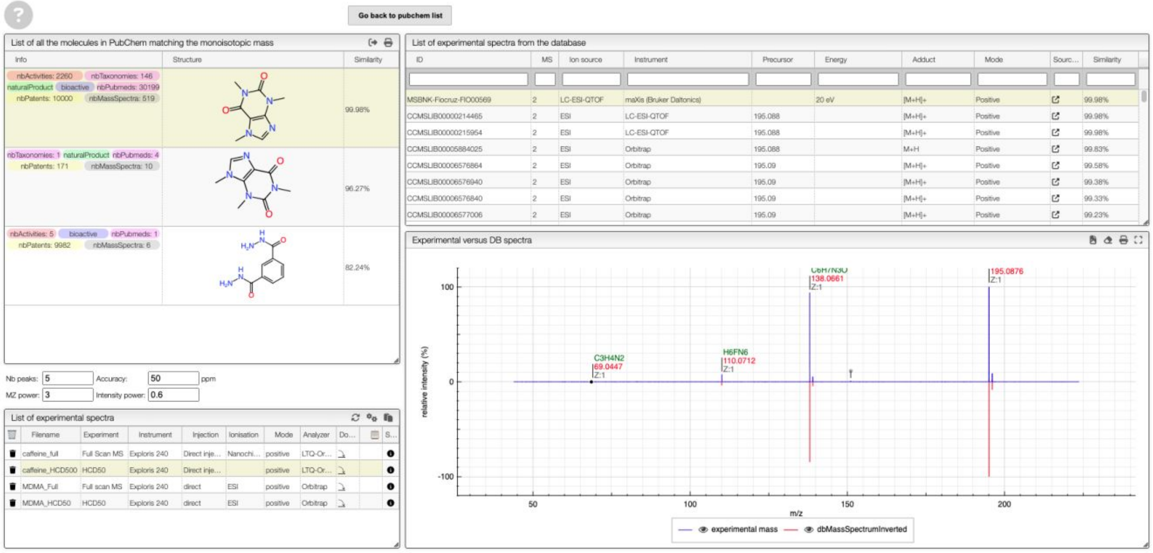

Figure S7: *MS/MS spectral database matching results showing cosine similarity scores for top candidate structures: Caffeine (99.98%), Isocaffeine (96.27%), and 1,3-Benzenedicarboxylic acid, dihydrazide (82.24%).*

Conclusion

Through a systematic dereplication workflow, the unknown compound in the sample was successfully identified as Caffeine. The process began with accurate mass measurement, where the experimental MS1 peak at 195.0874 m/z and isotopic pattern similarity of 99.57% strongly supported the molecular formula $C_8H_{10}N_4O_2$.

Fragment matching further reinforced this assignment, with 96.08% of MS/MS fragments aligning with theoretical predictions for $C_8H_{10}N_4O_2$, compared to only 0.82% for alternative candidates. The selection of diagnostic fragment ions (69.0447, 110.0711, and 138.0661 m/z) led to the hypothesis of an Imidazole substructure, found in 19 out of 26 database hits.

In the PubChem Tab, the formula $C_8H_{10}N_4O_2$ was associated with 2,028 known structures, 23 of which were bioactive. Filtering these based on the Imidazole substructure reduced the list to three likely candidates: Caffeine, Enprofylline, and 8-Methyltheophylline.

Finally, MS/MS spectral matching using the HCD 50 spectrum yielded a cosine similarity of 99.98% with Caffeine, while lower similarity or lack of spectral data was observed for the other candidates.

Altogether, the combination of high-resolution mass data, fragment analysis, database filtering, and spectral similarity scoring provided consistent and converging evidence for the identification of the compound as Caffeine.
